# Modulation of Nanowire Emitter Arrays Using Micro-LED Technology

**DOI:** 10.1021/acsnano.5c00474

**Published:** 2025-04-17

**Authors:** Zhongyi Xia, Dimitars Jevtics, Benoit Jack Eloi Guilhabert, Jonathan J. D. McKendry, Qian Gao, Hark Hoe Tan, Chennupati Jagadish, Martin D. Dawson, Michael John Strain

**Affiliations:** † Institute of Photonics, Department of Physics, University of Strathclyde, Glasgow G1 1RD Scotland, U.K.; ‡ ARC Centre of Excellence for Transformative Meta-Optical Systems, Department of Electronic Materials Engineering, Research School of Physics, The Australian National University, Canberra, ACT, 2600 Australian Capital Territory, Australia

**Keywords:** Nanowires, Transfer-Printing, micro-LEDs, Nanophotonics

## Abstract

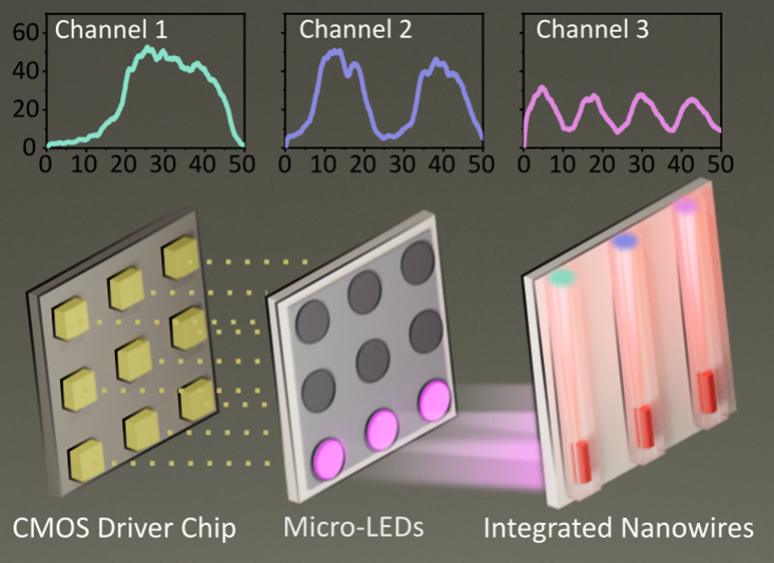

A scalable excitation platform for nanophotonic emitters using individually addressable micro-LED-on-CMOS arrays is presented. Heterogeneous integration by transfer printing of semiconductor nanowires was used for the deterministic assembly of the infrared emitters embedded in polymer optical waveguides with a high yield and positional accuracy. Direct optical pumping of these emitters is demonstrated using micro-LED pixels as a source, with optical modulation (on–off keying) measured up to 150 MHz. A micro-LED-on-CMOS array of pump sources was employed to demonstrate individual control of multiple waveguide-coupled nanowire emitters in parallel, enabling future large-scale photonic integrated circuit applications.

## Introduction

Light generation and modulation at the nanoscale have attracted significant attention in recent years due to the availability of a broad range of advanced materials and the growing maturity of heterogeneous integration techniques.^1−3^ Quasi-one-dimensional geometries, such as nanowires,^4−10^ nanopillars,^11^ nanobeam cavities,^12^ or cantilever-based emitters,^13^ are attractive solutions for on-chip emitters with efficient light coupling to waveguide platforms. The micro- or nano-dimensions of these emitters will play a crucial role in the next generation of nanophotonics, due to their low emission threshold, high optical confinement, the ability to tune emission wavelengths, and importantly, the ability to directly integrate these emitters with other photonic technologies, such as waveguides, antennas, or grating structures.^14,15^

The scalability of these devices from single-device demonstrations toward on-chip systems requires advances in two key areas: (1) the controllable integration of multiple emitters with existing chip-scale platforms and (2) the excitation of these devices using electronically controllable mechanisms that enable individual, high-speed addressing of multiple emitters in parallel.

The fabrication and integration of nanowire emitters is typically based on the transfer of these devices from their growth substrate onto a receiver substrate, either with existing optical structures^4,16^ or where post-transfer fabrication processes are used to pattern waveguide circuitry around the transferred nanowires.^5,6^ The techniques of heterogeneous integration, such as microtransfer printing,^17^ optical tweezers,^18^ and microprobe-based integration techniques,^8,10^ are among the most popular for the integration of this geometry of devices into photonic systems,^1^ allowing for the use of large-scale device population prescreening^19^ and the integration of devices into waveguides^4,5,10^ to move toward engineered systems-on-a-chip. The demonstration of multimaterial NW devices integrated on-chip has also been demonstrated, making use of the compact device footprints.^20−22^

The second challenge is that of scalable optical excitation of multiple nanowire sources in parallel, which is typically limited by the operation of continuous-wave or pulsed laser sources, which are generally used for their excitation. Laser pumps have a significant advantage over other means of excitation due to their coherent light emission, high peak power, and, where required, ultrashort pulses (reaching femtosecond time scales), making them invaluable for fundamental device and materials studies. However, laser spot excitation in microscope pumping arrangements limits optical addressing to single devices or clusters of photonic emitters at a time. Spatial light modulator (SLM) technology can be used to multiplex optical pump beams on a single substrate,^23^ but the individual modulation of each subspot, beyond the common pulsed mode excitation, is limited to the switching bandwidth of the SLM device, which is in the ∼10^4^ Hz range for current digital mirror device systems.^24^ Micro-LED-on-CMOS technology, on the other hand, has been rapidly advancing in functionality and scale over the past decade for applications in telecommunications,^25^ imaging,^26^ and displays.^27^ Recent work from our group demonstrated a 128 × 128-pixel micro-LED-on-CMOS display with nanosecond pulsing rates, independent pixel control at frame rates up to 0.5 Mfps, and 5 bit brightness control.^28^

## Results and Discussion

In this work, we demonstrate the deterministic integration of multiple nanowire devices into waveguide arrays on-chip and the individual control of these nanowire emitters using an arrayed violet micro-LED-on-CMOS pump source.^29^ By using electronically controllable LED array sources, we show individual addressing of multiple emitters in parallel and the route toward building scalable emitter systems for photonic integrated circuitry. The nanowire emitters used in this work are InP nanowires with a nominal diameter of ∼660 nm and lengths in the range of 6 μm, see refs (30,31) for further information. The devices were fabricated using a bottom-up approach, with a central emission wavelength at around 860 nm^17^ and high quantum efficiency, not only making them particularly bright, even at low excitation powers, but also ensuring their efficient absorption of the micro-LED light. These semiconductor emitters are robust and do not degrade during the transfer process, or under solvent treatment, which is advantageous for a multistep fabrication process.^5,19^

**Figure 1:**
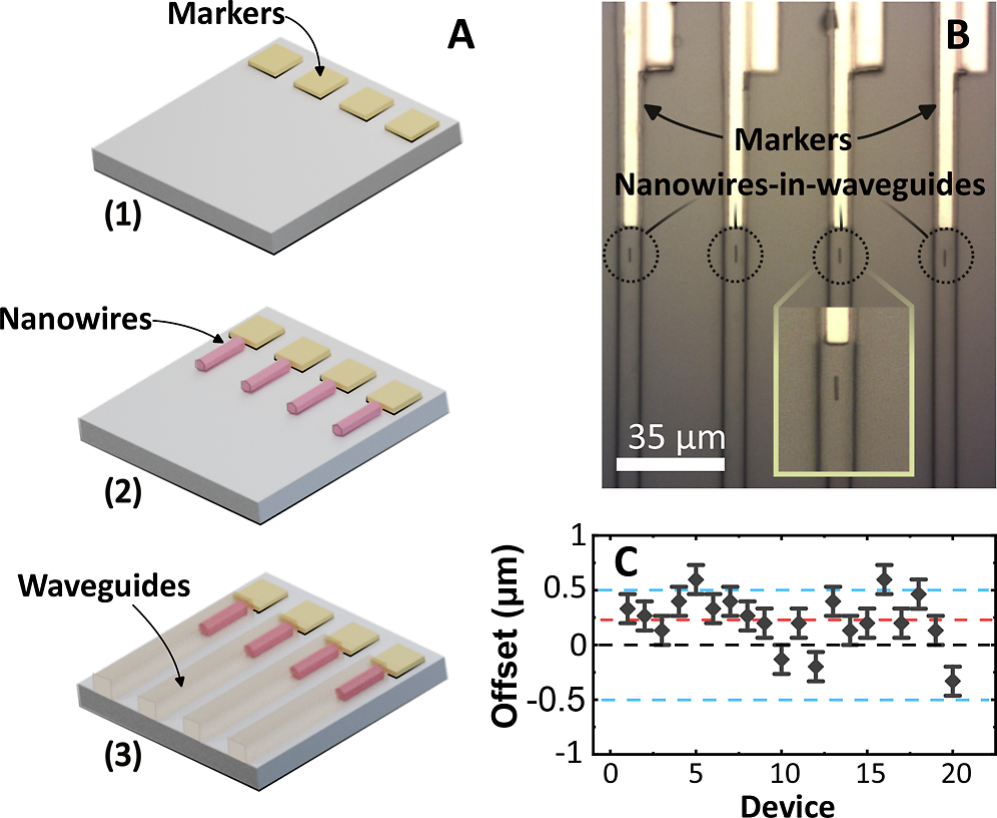
(A) Schematic flow diagram showing the fabrication process of embedding nanowire devices into polymeric waveguides. (B) Plan view image of the processed nanowire-in-waveguide arrays. The inset shows an enlarged image of an embedded-into-waveguide nanowire. (C) A lateral offset scatter plot of the 20 nanowire-in-waveguide devices, with blue lines indicating ±500 nm and the red line showing the estimated average offset of 228 nm.

After their growth, the nanowires were transferred to a host polymer (polydimethylsiloxane, PDMS) using a mechanical transfer process.^19^ The target nanowire devices were then individually selected for further printing into the waveguides. A schematic of the fabrication process is shown in Figure 1A, where a borosilicate glass was used as a substrate, and metal marker structures were fabricated using direct write laser lithography and a metal lift-off process of a Ti/Au (50:200 nm) bilayer. A heterogeneous integration technique was then used to pick and place individual nanowires from the host PDMS substrate onto the glass substrate aligned to the central axis of the metal markers using previously developed alignment techniques.^32^ After the nanowires were printed, a second laser lithography process, into a 4 μm-thick SU-8 polymer resist, was carried out to define the optical waveguide structures. This process resulted in nanowires fully embedded into the polymer waveguide, enhancing the optical mode coupling efficiency between the structures,^5,6^ compared with end-fire or laterally coupled geometries.^4^ In total, 20 nanowire-in-waveguide devices were fabricated in a linear array with the waveguide spacing matching the projected pitch of 33 μm of the elements of the micro-LED array. A brightfield image of the embedded-nanowire array section is shown in Figure 1B. The average offset of the nanowire major axis from the center of the waveguides was estimated to be 228 nm following the same estimation technique described in ref (32) and using a high-magnification optical microscope, as shown in the scatter plot in Figure 1C.

**Figure 2:**
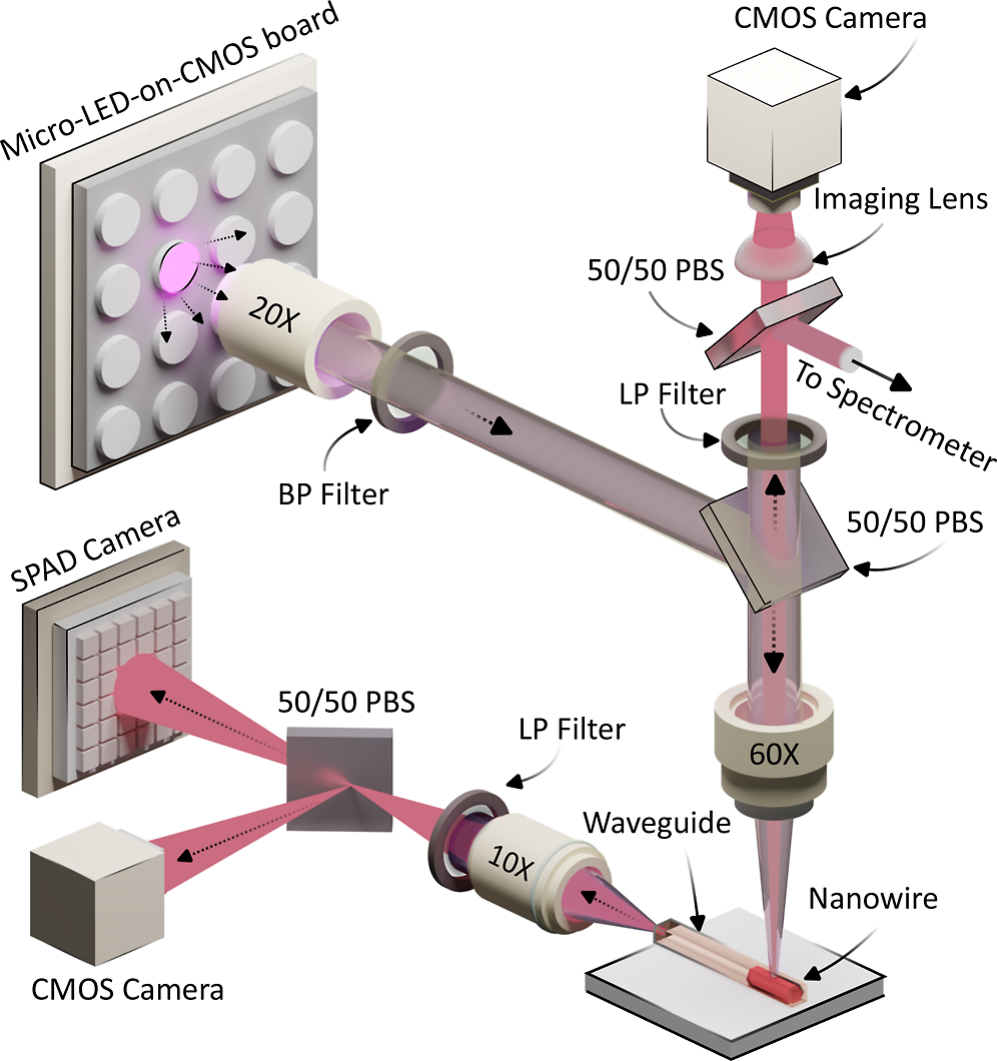
Schematic diagram showing the μ-photoluminescence setup used for the optical excitation of nanowire emitters using micro-LED-on-CMOS technology.

**Figure 3:**
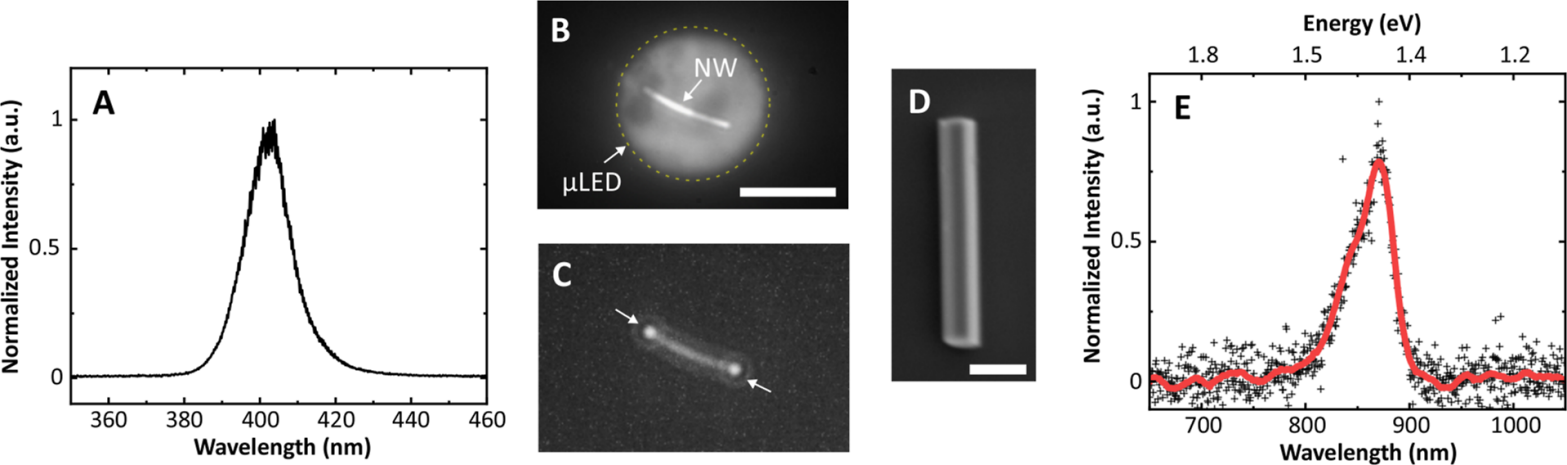
(A) Spectrum of a single micro-LED pixel. (B) Plan view optical image showing the emission of a single micro-LED pixel projected onto a single nanowire emitter on a quartz substrate. Scale bar = 12 μm. (C) Filtered image showing emission patterns coming out of the nanowire facets, excited with the projected micro-LED. (D) SEM micrograph of a representative InP nanowire emitter on the Si substrate. Scale bar = 1 μm. (E) Spectrum from a single InP nanowire emitter on quartz under micro-LED optical excitation.

The micro-LED-on-CMOS chip μ-photoluminescence (μ-PL) setup diagram for optical excitation and modulation of nanowire devices is shown in Figure 2. The micro-LED-on-CMOS chip consists of a 16 × 16 active pixel array, where the diameter of the individual circular pixels is 72 μm, on a pitch of 100 μm. The optical power used here from an individual pixel in continuous-wave mode is of the order of 1 mW, with a peak emission wavelength of 402 nm,^29^ as shown in Figure 3A. The cutoff frequency for the full on–off keying of the micro-LED pixels was measured at ∼120 MHz, but this was limited here by the CMOS drive electronics since the micro-LEDs have a bandwidth of up to 100s MHz.^29^ The micro-LED-on-CMOS array is mounted on a 3-axis stage allowing for fine alignment control with free-space optics. For the optical projection of micro-LED patterns onto a sample, a set of lenses is used to couple the projected light to the back-aperture of a 60× optical objective. An optical bandpass filter (Thorlabs, FBH400-40) was also used to filter out unwanted defect-related photoluminescence of GaN-based micro-LEDs.^33^ A high quantum efficiency CMOS camera is used to image the emitted light from the micro-LED that is back-reflected through the optical column for alignment to the on-chip nanowire-in-waveguide devices. An edge detection setup is used to capture the light emitted from the end facet of the optical waveguides, imaging through a 10× objective lens. The facet-coupled light is then split and filtered using a 50:50 nonpolarizing beamsplitter (BS) and long-pass filter (Thorlabs, FELH0800) into a high-sensitivity monochromatic CMOS camera and single-photon avalanche diode (SPAD) detector (PhotonForce32). The former is used for alignment and imaging purposes and the latter for time domain light modulation measurements.

To characterize the nanowire emission properties, an array of nanowires was mass transferred onto a quartz disk substrate (diameter 12 mm, thickness 2 mm) using a PDMS slab, as described in ref (19). The total micro-LED fluence (irradiance) of ∼280 mW/mm^2^ was projected onto a sample, and an image of the micro-LED spot overlapping a single nanowire device is shown in Figure 3B. From spatial overlap considerations, an estimated ∼1% of the projected micro-LED illumination is incident on the single nanowire device. The spatial mismatch of the micro-LED pixel and the emitter geometry could be improved by using shaped micro-LED apertures to match the profile of nanowire devices. Airy disks at each of the nanowire’s facets are clearly visible on the pump-filtered micrograph in Figure 3C. An SEM image of a representative InP nanowire emitter embedded into waveguides (in Figure 1) is shown in Figure 3D. The photoluminescence (PL) of a single NW was recorded using a focusing lens (*f* = 35 mm) placed in the path labeled “to spectrometer” in Figure 2. The spectrometer was a fiber-coupled spectrometer (Avantes, Multichannel Spectrometer) with a 24 s integration time and 4 averaging scans to improve the signal-to-noise ratio. Figure 3E shows a measured nanowire emission spectrum taken under these low-light micro-LED illumination conditions, which is characteristic of the expected nanowire spontaneous emission.^30^

**Figure 4:**
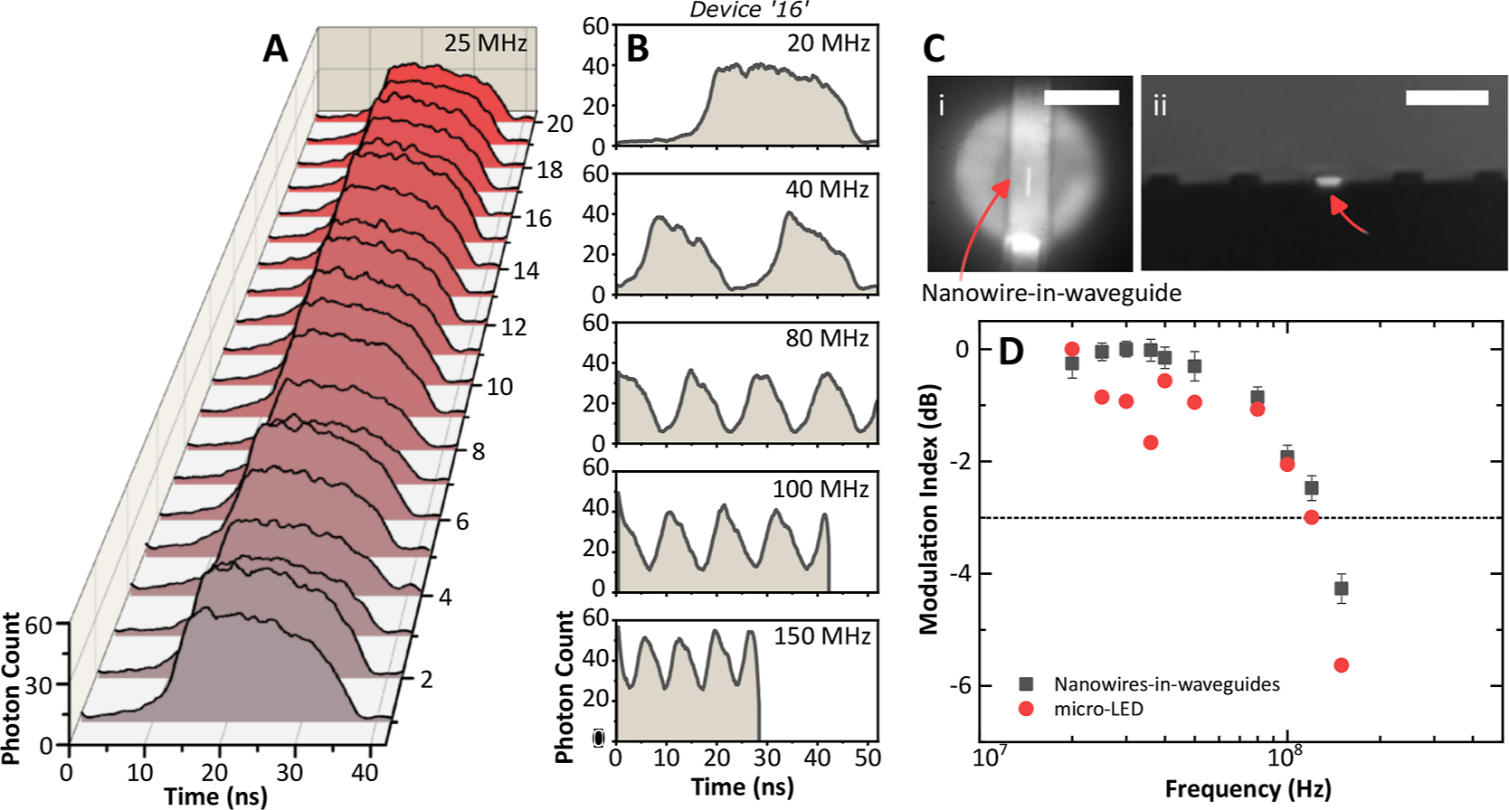
(A) Diagram showing time-domain measurements at a modulation frequency of 25 MHz for 20 waveguide-integrated nanowire emitters. (B) Time-domain measurements of device “16” at modulation frequencies of 20, 40, 80, 100, and 150 MHz, respectively. (C) (i) Brightfield micrograph showing the projected single pixel micro-LED emission exciting a single waveguide integrated nanowire and (ii) waveguide facet showing the light from the nanowire. Scale bars in (i) = 12 μm and in (ii) = 35 μm. (D) Cutoff frequency plot showing the modulation index for both nanowire-in-waveguide emitters (mean value for 20 devices) and micro-LED pixel.

As described previously, target nanowire devices were embedded into polymeric SU-8 waveguides using a multistage microfabrication process. Each embedded nanowire emitter was optically excited and modulated (on–off keying) by using the micro-LED-on-CMOS system. An external frequency source (Liquid Instruments, Moku/Pro) was connected to the CMOS board, allowing one to sweep between the excitation frequencies. Using the edge-detection setup, light collected at the facet of a nanowire-waveguide-coupled device was captured by using a SPAD camera. Due to the acquisition window of the SPAD camera, frequencies ranging from 20 to 150 MHz were selected, corresponding to 50–6.67 ns periods, respectively. To collect time-domain measurements of the modulated nanowire devices, both the micro-LED-on-CMOS board and a SPAD camera were connected to the outputs of an external frequency generator for triggering the data collection; the frequency and phase of both outputs were synchronized. The modulation characteristics of all 20 channels of the nanowire-in-waveguide devices were first measured independently by using the SPAD camera. Figure 4A shows a captured signal from all 20 nanowire-in-waveguide channels optically modulated at 25 MHz. The repeatability of the heterogeneous technique and integration into waveguides ensure that all 20 devices have comparable performance in terms of photon counts. The data was processed using a Savitzky–Golay digital filter with a third-order polynomial and a window size of 53 samples for all modulation plots. Figure 4B shows modulation measurements at different frequencies (20, 40, 80, 100, and 150 MHz) for a representative device ‘16’, demonstrating on–off keying operation beyond the 3 dB cutoff frequency of the micro-LED devices. The top view microscope image in [Figure 4C(i)] shows a micro-LED projected onto a waveguide with a nanowire device located inside the waveguide structure. A side view of the edge of the sample showing output light at 20 MHz from the excited nanowire inside the waveguide is shown in [Figure 4C(ii)]. The measured cutoff frequencies, see Figure 4D, of both micro-LED-on-CMOS board and embedded nanowire-in-waveguide devices (mean values) show that the latter follows the temporal envelope of the micro-LED emission, highlighting that the speed limitation originates with the pump as expected.^10^ The modulation index is defined as , where *P*_max_ and *P*_min_ are maximum and minimum intensity values, respectively.^10^ The nanowire devices exhibit slightly higher modulation index values as compared to the micro-LED devices. This is likely related to the amplified spontaneous emission operation of the nanowires, which provides gain as a function of optical pump level, improving the signal-to-noise ratio. Furthermore, during the measurement of the low-light waveguide-coupled experiments, we observed luminescence from the SU-8 waveguide;^34^ however, it was characterized to be ∼28 times lower than the coupled emission from the integrated nanowire under the same optical pump conditions and using an 800 nm optical long-pass filter (Thorlabs, FELH0800).

**Figure 5:**
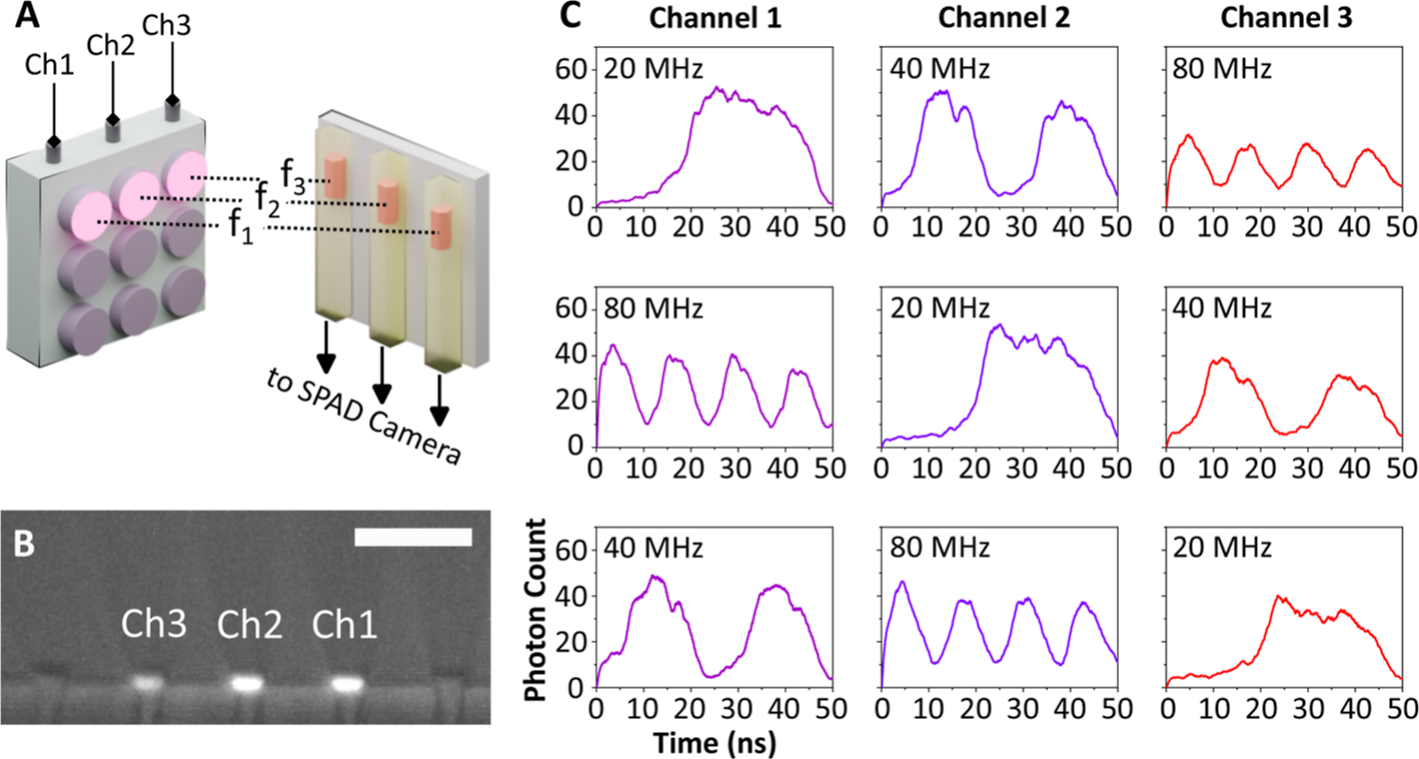
(A) A schematic diagram showing each of three adjacent and independently modulated micro-LED pixels separately exciting each of three adjacent embedded-into-waveguide nanowire emitters. (B) Side view of the edge of the waveguide sample showing output light from the three excited nanowires inside waveguides. Scale bar = 35 μm. (C) Time-domain modulation measurements (on–off keying) simultaneously collected from three waveguide-embedded nanowires at frequencies of 80, 40, and 20 MHz, each horizontal panel representing a different configuration of modulation frequency.

To demonstrate the device excitation multiplexing capability, three adjacent micro-LEDs were projected onto three individual nanowire-in-waveguide devices at different frequencies (*f*_1_, *f*_2_, *f*_3_), as shown schematically in Figure 5A. By using free-space optical coupling from the micro-LED array, we can project three pixels without a significant power reduction of the excitation from the single pixel case. A different set of optics with a larger field of view could significantly increase the number of projected micro-LEDs with a penalty on the optical throughput. The SPAD camera, consisting of a 32 × 32 array of pixels and using a 10× optical objective at the waveguide facet, allows imaging of the three waveguides in parallel. Each micro-LED pixel was independently modulated at 20, 40, and 80 MHz in different configurations. Figure 5B shows three waveguide facets, each representing individual coupled nanowire emitters modulated at frequencies of 20, 40, and 80 MHz for channels 1, 2, and 3, respectively. Figure 5C shows the results of independent frequency modulation of the three different channels of the sample with the individual waveguide channels measured on the SPAD array as isolated fields of view, in this case, single pixels of the SPAD array. From photon count levels corresponding to each waveguide, it is clear that the waveguides have comparable signal levels, and the nanowire devices follow the modulated on–off keying of the micro-LEDs.

## Conclusions

In summary, we have demonstrated the ability to create uniform arrays of nanowire-in-waveguide devices using a heterogeneous integration technique and post-transfer fabrication of polymer waveguides. The high yield of the device fabrication process is important for system scalability and enables the preselection of emitters with similar operating characteristics, or with advanced device binning, out of ensembles of nanowires. Optical pumping of nanowire emitters using micro-LED sources is demonstrated for the first time to the best of our knowledge. The use of micro-LED-on-CMOS arrays as pump sources shows the capability for scalable, high-speed optical pumping of multiple emitters in parallel, and experimental results show waveguide-coupled optical signals in the 10s of MHz range, limited in this case by the current CMOS driver chip. Reduction of the waveguide width and pitch would allow individual optical excitation of up to nine devices within a 100 × 100 μm^2^ area under the same micro-LED projection configuration. The next stage of this work will be to integrate the optical pump system and nanowire-in-waveguide devices into a single package, creating a compact system and removing the need for projection optics.

## Methods

### Nanowire Growth

The semiconductor InP nanowires of this work were grown by selective area metal–organic vapor phase epitaxy (MOPVE). At first, a 30 nm thick layer of SiO_2_ was deposited on the InP (111)A substrate by plasma-enhanced chemical vapor deposition. A hexagonal array of circled features with a nominal diameter of 420 nm and a target device pitch of 1.5 μm was then patterned on the SiO_2_ layer using electron beam lithography. Prior to the nanowire growth, the SiO_2_ layer was removed by wet chemical etching. Finally, nanowires of an average diameter of 660 nm were grown using MOPVE. A full description of the nanowire fabrication process can be found in.^30^

### Waveguide Fabrication

A layer of positive photoresist (Shipley series, S1818) was spin-coated onto a borosilicate glass to a thickness of 1.5 μm. The sample was then soft-baked on a hot place at 115 °C for 1 min. Fiducial patterns (markers) were laser-written (Heidelberg DWL 66+) and developed using a photoresist developer (Microdeveloper) mixed with deionized water (1:1 ratio). Using an electron-beam-based evaporation tool (FerroTec Temescal FC-2000), a Titanium–Gold metal stack (50 and 200 nm, respectively) was deposited onto the sample. After metal lift-off using an 1165 remover, the sample was moved onto the transfer-printing machine for the nanowire alignment and integration process. After the nanowire integration, a layer of epoxy-based negative photoresist (SU-8 6005) was spin-coated onto the sample to a thickness of 4 μm. The waveguides were then laser-written directly in the SU-8 and developed using propylene-glycol-methyl-ether-acetate (PGMEA).

### Transfer-Printing of Nanowire Emitters

The transfer printing of semiconductor nanowires was performed using a flat PDMS microstamp head with a surface area of 10 × 30 μm^2^. For the nanowire capture, the microstamp was aligned with a target nanowire before being brought into contact and picked up from the substrate using a rapid vertical retraction. The nanowires were then aligned (for the nanowire alignment description see^32^) with the markers on the waveguide sample. Nanowire emitters then were individually printed (integrated) onto the target locations next to the fiducial markers. The full nanowire transfer printing process is reported in.^17^
